# Millennium Development Goals progress: a perspective from sub-Saharan Africa

**DOI:** 10.1136/archdischild-2013-305747

**Published:** 2015-02

**Authors:** Mike English, Rex English, Atti English

**Affiliations:** 1 KEMRI-Wellcome Trust Research Programme, Nairobi, Kenya; 2 Nuffield Department of Medicine, University of Oxford, Oxford, UK; 3 Clare College, Cambridge University, Cambridge, UK; 4 Pembroke College, Cambridge University, Cambridge, UK

**Keywords:** Tropical Paediatrics, Comm Child Health, Epidemiology, General Paediatrics

## Abstract

Sub-Saharan Africa is a highly diverse geo-political region. Any brief discussion of the progress made over the last 15 years towards the Millennium Development Goals (MDGs) will therefore not do justice to the true complexity of context and events. Our focus will be MDG4—to reduce child mortality by 66% from 1990 levels. We will touch briefly on MDG1, to eradicate extreme poverty and hunger, MDG2, to achieve universal primary education, and MDG5, to improve maternal health, which are inextricably linked with child well-being. We will also draw on an eclectic mix of additional global indicators. Acknowledging the limitations of this approach, we first offer a summary of expected progress and then point to debates on future goals.

## Progress made

The latest data available in 2014 on progress in meeting Millennium Development Goal (MDG) targets for goals 1, 2, 4 and 5 are summarised in table 1 (data derived from refs. 1–3). As has been articulated in detail elsewhere, the biggest gains have been made in reducing infant and child mortality, making persistently high neonatal mortality now a major concern.^1^ ^3^ There are some differences in progress across regions. Countries in East, West and Southern Africa have in general made better progress than those in Central Africa. However, there are notable gaps in data. Data are completely inadequate to evaluate progress across the four goals listed in table 1 for 7/48 countries, and there are major gaps in data for 16/48 countries (a theme we will return to later). One apparently obvious association with either lack of data or poor progress is major conflict. Over the past decade, 13/48 countries in sub-Saharan Africa (SSA) have been affected by civil wars resulting in major population displacement (this excludes the much larger number of more localised conflicts).

**Table 1:** A summary of progress made by sub-Saharan African countries towards Millennium Development Goals 1, 2, 4 and 5 (data drawn from references 1–3)

Goal	Progress indicator	Countries meeting indicator target*
MDG4	95–100% progress towards target	8/43
	50–95% progress towards target	19/43
	<50% progress towards target	16/43
MDG1	No progress in last decade	8/26
MDG2	<50% progress towards target	5/43
MDG5	<50% progress towards target	18/43
Aggregated: MDG1, MDG2, MDG4 and MDG5	Insufficient progress in 3 or 4 of these goals	23/25
	Likely to achieve all 4 MDG goals	0/25

*The denominator reflects the number of countries for which data were available, the numerator the number of countries achieving the indicator standard.

MDG, Millennium Development Goal.

Where national governments have taken the lead, complementary bilateral, multi-national and philanthropic efforts have probably been significant in helping countries make progress. Vaccine coverage has improved and new vaccines against hepatitis B, *Haemophilus influenzae* type B and pneumococcus have been deployed, with rotavirus vaccine programmes also now being initiated. New anti-malarials have been introduced and the mass distribution of insecticide-treated bednets undertaken. Vitamin and micronutrient supplementation strategies have been initiated and all this has been allied to improved access to primary care, clean water and sanitation. However, there is still plenty to be done to support the delivery of such basic interventions and to ensure full and equitable access in nearly all of these. However, the era of identifying specific interventions that by themselves have a major impact on child survival is likely drawing to a close.

## Future development goals

Recent debate has highlighted the principle of universal health coverage whereby everybody obtains the health services they need without experiencing financial hardship. This requires a strong, adequately financed, efficient and well-run health system, access to essential medicines and technologies, and sufficient well-trained and motivated health workers. These will be particularly needed as SSA countries face the challenges posed by non-communicable and lifestyle-related disease and the ongoing challenge of neglected tropical diseases. Discussions on the post-2015 successors to the MDGs, the new Sustainable Development Goals (SDG), recognise development inter-relatedness even more explicitly, being aware that health gains are co-produced by progress in and good governance of sectors as diverse as agriculture, industry, education and the environment. The SDGs are therefore the concern of countries, regional bodies and many United Nations organisations.

As we enter this new era, how well equipped are SSA countries to make progress or continue extending gains? Addressing this question in detail is beyond the scope of this commentary, but some global indicators point to important challenges. For example, official development assistance (ODA) for health per capita/year in the WHO African Region increased from US$2.7 in 2002 to US$9.8 in 2010. More than 50% of this assistance supported efforts to make progress against HIV, malaria and tuberculosis (MDG6) by 2010 (data from reference 3). At the same time, governments’ spending on health has increased, although only 6/46 countries have met their Abuja target^3^ of 15% of their expenditure. Thus, in 2012 donor assistance still represented >30% of total health spending in 18/46 SSA countries, while out-of-pocket (personal) spending accounted for >30% in 29/46 countries (data from ref. 3). Reflecting long-standing limited investment in health services, 0/34 and 3/33 countries (South Africa, Lesotho and Namibia) had at least one physician or two nurses/midwives per 1000 population, respectively,^3^ densities felt to be too low to deliver basic services. At the same time, 20/44 countries are ranked in the bottom 50 of 162 countries represented in the Global Peace Index and 23/48 in the bottom 50 of 177 countries in the Corruption Perception Index. Thus, although there is optimism that African economies may grow at annual rates of approximately 5%, this may not result in efficiently delivered and effective coverage by quality health services. Furthermore, available data for 23/34 SSA countries suggest substantial inequity (GINI coefficient ≥0.4; the GINI coefficient ranges from 0, perfect equity, to 1, absolute inequity, with a value of 0.4 being considered the international alert line)), highlighting that the poor may benefit least from health investments, thus limiting overall health gains.^3^

## Information: measuring progress, promoting accountability and sharing learning

So, could ambitions to improve healthcare systems yield results? History may be instructive. By the 1920s, Norway had achieved under-one mortality rates of approximately 40/1000, a rate that fell to less than 20/1000 by the start of the Second World War. Sri Lanka had achieved infant mortality rates comparable to those in some of today's best performing middle-income countries by 1960.^4^ Clearly, in both cases this was without modern vaccines, medicines or technologies. In the case of Sri Lanka, progress was based on providing ready access to professionally led health services for the entire population.^4^ Also worth noting is the ability of such countries to measure their progress at scale 50–80 years ago and more. In SSA, only one country captures >50% deaths in a vital registration system (South Africa) and in many countries such systems hardly exist.^5^ Thus, although the methods for estimating health outcomes have become much more sophisticated, those available have wide limits of precision for outcomes such as maternal mortality and rarely provide for sub-national tracking. This undermines the ability of citizens to hold their governments accountable for their stewardship of health systems, a fact relatively recently acknowledged in the creation of the WHO's Accountability Commission.^5^ Continued investments in health systems should ensure that high quality data collection and analysis are at their centre. These data, allied to other forms of evidence, are also required in order to inform SSA countries’ health investment choices, to examine their consequences, and to evaluate innovations in health system design rather than simply mimic high-income countries’ models of care. Shared learning from successes could then help accelerate the pace of health system improvements, accepting that learning from efforts to tackle the ‘wicked problems’—problems that are difficult or impossible to solve because of incomplete, contradictory and changing requirements—of health systems is in itself a challenge.
